# Low-affinity anti-HER2-CAR T cells efficiently eradicate HER2^+^ and HER2^low^ cancer cells with reduced toxicity on healthy tissue

**DOI:** 10.3389/fbioe.2026.1895507

**Published:** 2026-10-12

**Authors:** Leonard Knoedler, Konstantin Herfeld, Julian N. Scherer, Kaiji Fan, Dhyani Shah, Sascha Goettert, Luisa Becker, Iria Lloshi, Suqi Li, Dennis C. Harrer, Matthias A. Fante, Tobias Wertheimer, Roland Schelker, Severin Guetter, Misbah Tariq, Omer Khalid, Alexander Scheiter, Wolfgang Herr, Christian Schmidl, Moritz Jesinghaus, Markus Tschurtschenthaler, Hendrik Poeck, Markus Perl

**Affiliations:** 1 Department of Internal Medicine III, Hematology & Internal Oncology, University Hospital Regensburg, Regensburg, Germany; 2 Division of Plastic and Reconstructive Surgery, Cedars-Sinai Medical Center, Los Angeles, CA, United States; 3 Department of Oral and Maxillofacial Surgery, Charité–Universitätsmedizin Berlin, Berlin, Germany; 4 Leibniz Institute for Immunotherapy (LIT), Regensburg, Germany; 5 Division of Hematology and Oncology, Department of Medicine, Freiburg University Medical Center, Albert-Ludwigs-University, Freiburg, Germany; 6 Institute of Pathology, University Hospital Regensburg, Regensburg, Germany; 7 Institute of Pathology, Phillips University Marburg, Marburg, Germany; 8 School of Medicine, Center for Translational Cancer Research (TranslaTUM), Technical University of Munich, Munich, Germany; 9 German Cancer Consortium (DKTK), Partner-site Munich and German Cancer Research Center (DKFZ), Heidelberg, Germany; 10 Chair of Translational Cancer Research and Institute of Experimental Cancer Therapy, Klinikum Rechts der Isar, TUM School of Medicine and Health, Technical University of Munich, Munich, Germany; 11 Bavarian Cancer Research Center (BZKF), Regensburg, Germany

**Keywords:** anti-HER2, bioluminescence imaging, CAR T cells, chimeric antigen receptor, organoids, solid tumor, systemic tumor burden

## Abstract

Chimeric antigen receptor (CAR) T cell therapy is considered a therapeutic breakthrough in hematological malignancies. However, its translation to metastasized solid tumors remains limited by the paucity of “druggable” targets and the limited therapeutic potency of the cell therapeutics in solid cancers. Human epidermal growth factor receptor 2 (HER2), a promising target for treatment of metastasized solid cancers, is amplified in around 3% (HER2^+^ cancer) and overexpressed without amplification in ∼40% (HER2^low^ cancer) of all carcinomas, yet the physiological expression of HER2 on healthy tissues poses the risk of on-target off-tumor effects. To address this, we utilized low-affinity (LA) Anti-HER2-CAR T cells. These CAR T cells exhibited efficient lysis of HER2^+^ SKOV3 ovarian cancer cells even at low effector-to-target ratios. Following stimulation through cancer cells, LA Anti-HER2-CAR T cells showed rapid proliferation and elevation of pro-inflammatory cytokines markers. Low numbers of CAR T cells were sufficient to induce durable complete remissions of systemically seeded ovarian cancer in NOD-*Prkdc* ^*scid*^ *-IL2rg* ^*Tm1*^/Rj mice. LA Anti-HER2 CAR T cells also exhibited tumor regression and lasting tumor control in a subcutaneous *in vivo* model of HER2^low^ LS174T cells. Additionally, the LA and high-affinity (HA) CAR T cells could effectively combat HER2^low^ human colorectal cancer (CRC) cell lines and primary CRC organoids, while LA Anti-HER2-CAR T cells showed a trend-wise reduced lysis of healthy colon organoids. Our results suggest that LA Anti-HER2-CAR T cells provide a novel treatment option for HER2-overexpressing advanced systemic tumors. Importantly, they effectively distinguished between healthy colon tissue and HER2^low^ cancer, broadening the scope of HER2-directed CAR T cells to this frequent cancer subset.

## Graphical Abstract

**Figure:**
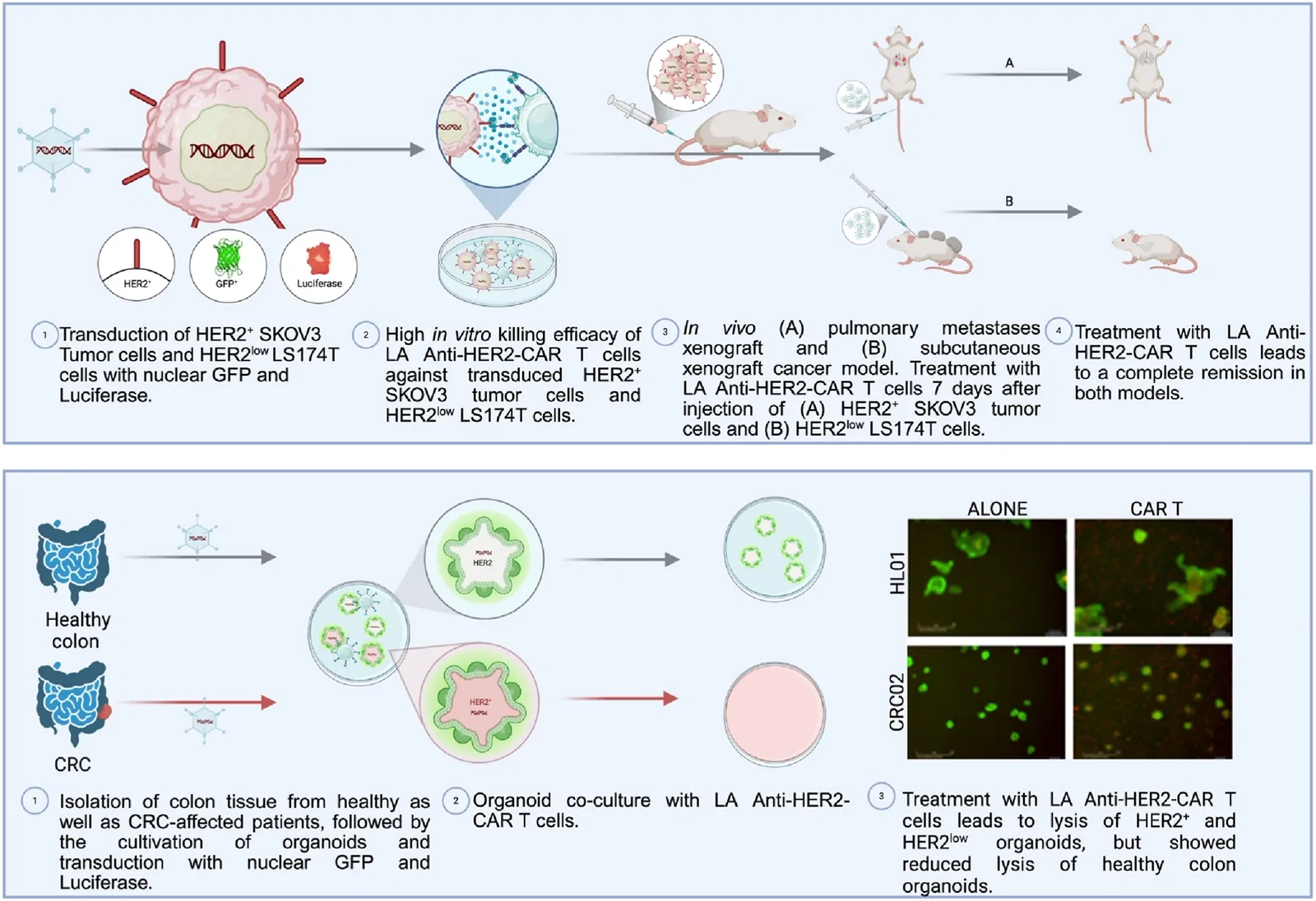
GFP, Green Fluorescent Protein; CRC, Colorectal Cancer; LA, Low-affinity.

## Introduction

Solid cancer account for approximately 90% of adult human cancers, affecting more than 800 million people worldwide (Siegel et al., 2024). Once metastasized to essential organs (e.g., lung, brain, liver, bone marrow), solid tumors become largely incurable, reducing 5-year survival rates by up to 70% across the four most common entities (breast, lung, prostate, colon) and driving high mortality among affected patients (Siegel et al., 2025; Piffko et al., 2022; Su et al., 2023).

Chimeric antigen receptor (CAR) T cell therapy–bioengineering T cells to target tumor surface antigens (e.g., BCMA, CD19) – represents a novel therapeutic option, especially in patients with treatment-resistant or recurrent systemic malignancies (Kauke-Navarro et al., 2023; Freitag et al., 2020). CAR T cells have been effective in treating different hematological malignancies such as acute lymphoblastic leukemia, diffuse large B-cell lymphoma and multiple myeloma, yet the tumor microenvironment and heterogeneity of solid tumors continue to limit its clinical success in this setting (Daei et al., 2023; Guzman et al., 2023).

The cellular surface receptor human epidermal growth factor receptor 2 (HER2), which drives tumor growth and malignancy, was among the first specific CAR targets in solid tumor treatment (Ahmed et al., 2015). 10%–20% of all breast cancer subtypes, and around 10% of gastric cancers exhibit a gene amplification of HER2, leading to a significant overexpression of the receptor (Krishnamurti and Silverman, 2014; Kanayama et al., 2018). This condition is associated with poor patient survival due to the receptor’s association with high levels of invasiveness and aggressiveness. The advent of specific Anti-HER2 antibodies like Trastuzumab in the late 1990s marked a turning point in addressing this issue. Beyond breast and gastric cancers, HER2 positivity linked with HER2 gene amplification is present in about 3% of epithelial tumors (Yan et al., 2015). However, around 40% of epithelial cancers such as colorectal carcinoma overexpress the receptor without its amplification. These tumors are classified as HER2^low^ tumors (Iqbal and Iqbal, 2014; Modi et al., 2022; Doi et al., 2017). Recently, Trastuzumab antibody-drug conjugates received EMA and FDA approvals to treat HER2^low^ tumors (Meric-Bernstam et al., 2024; Hurvitz et al., 2023). Yet, low levels of HER2 are also found in healthy epithelial tissue and, therefore, HER2-targeting therapies are associated with significant side effects, including pulmonary and gastrointestinal toxicities (Morgan et al., 2010; Rugo et al., 2023). Thus, various strategies have been developed to more effectively distinguish between HER2^+^ tumors (i.e., tumors with HER2 gene amplification) and healthy tissue. One such approach involves engineering the HER2-binding scFv to bind HER2 with substantially lower affinity. This strategy aims to fine-tune antigen density-based targeting through CAR T cells (Hernandez-Lopez et al., 2021; Shabaneh et al., 2024; Barden et al., 2024). For instance, LA Anti-HER2-CAR T cells exhibited less liver damage and lower systemic levels of Interferon gamma (IFN-γ) than HA CAR T cells while demonstrating superior antitumor efficacy activity against HER2^+^ cancers *in vivo* (Castellarin et al., 2020). Similarly, Liu et al. reported that LA Anti-HER2-CAR T exhibited robust antitumor efficacy comparable to HA cells but spared normal cells expressing physiologic target levels in a murine prostate cancer model (Liu et al., 2015). There is also limited clinical data that implicates Anti-HER2-CAR T cells with severe pulmonary toxicity in one study patient, although the impact of additional, potentially confounding factors (e.g., high dosage of 1 × 10^10^ CAR T cells) remains to be determined (Morgan et al., 2010).

Nonetheless, it remains to be elucidated whether reducing the HER2 affinity of the CAR ScFv against its target, effectively treats HER2^low^ tumors while sparing healthy tissue and avoiding toxicities. Additionally, there is a paucity of experimental models to simulate systemic tumor seeding and investigate therapeutic effects of CAR T cells on the systemic tumor burden.

Therefore, we established an *in vivo* model to visualize systemic seeding of HER2^+^ SKOV3 ovarian cancer cells over time and determine the response to previously developed LA Anti-HER2-CAR T cells in spreading as well as established HER2^low^ subcutaneous tumors (Hernandez-Lopez et al., 2021). We also investigated if LA Anti-HER2-CAR T cells are effective against primary HER2^low^ cancer organoids and spare healthy epithelium.

## Results

### A lentivirus for bicistronic expression of Luc2 and nuclear EGFP to sensitively track cancer cells

To test the effects of Anti-HER2-CAR T cells, we aimed to sensitively track both cancer cell viability and cell count *in vivo* and *in vitro*. We constructed a VSV-G pseudotyped lentivirus encoding for a humanized high-fidelity firefly luciferase (Luc2) and enhanced green fluorescent protein (EGFP) coupled to nuclear localisation sequences at both ends (Luc2-P2A-NLSEGFP) driven by the strong, constitutively expressed Human eukaryotic translation elongation factor 1 α1 (EF1A) promoter. The transgenes were separated by a self-cleaving P2A linker (Supplementary Material 1b). This design allows both, cell quantifications based on luminescence signals and precise cell counting in live microscopy as fluorescent nuclei can be automatically detected by conventional imaging software. After viral packaging, the virus was concentrated by ultracentrifugation.

Using this second generation lentivirus, we transduced human cell lines (SKOV3, LS174T), primary human colorectal cancer (CRC) and healthy large intestine organoids. On day 5 after transduction, cell lines, as well as CRC and colon organoids exhibited high EGFP expression in flow cytometry after transduction at a multiplicity of infection (MOI) of 5. Subsequentially, cells were sorted for EGFP expression utilizing flow cytometry and expanded afterwards.

EGFP and HER2 expression was confirmed by flow cytometry (Figures 1a,b). Importantly, transduced cells maintained transgene expression in serial flow cytometry and bioluminescence analyses over at least 30 passages and multiple freeze-thaw cycles (Figure 1a, gating strategy in Supplementary Material 2).

**FIGURE 1:**
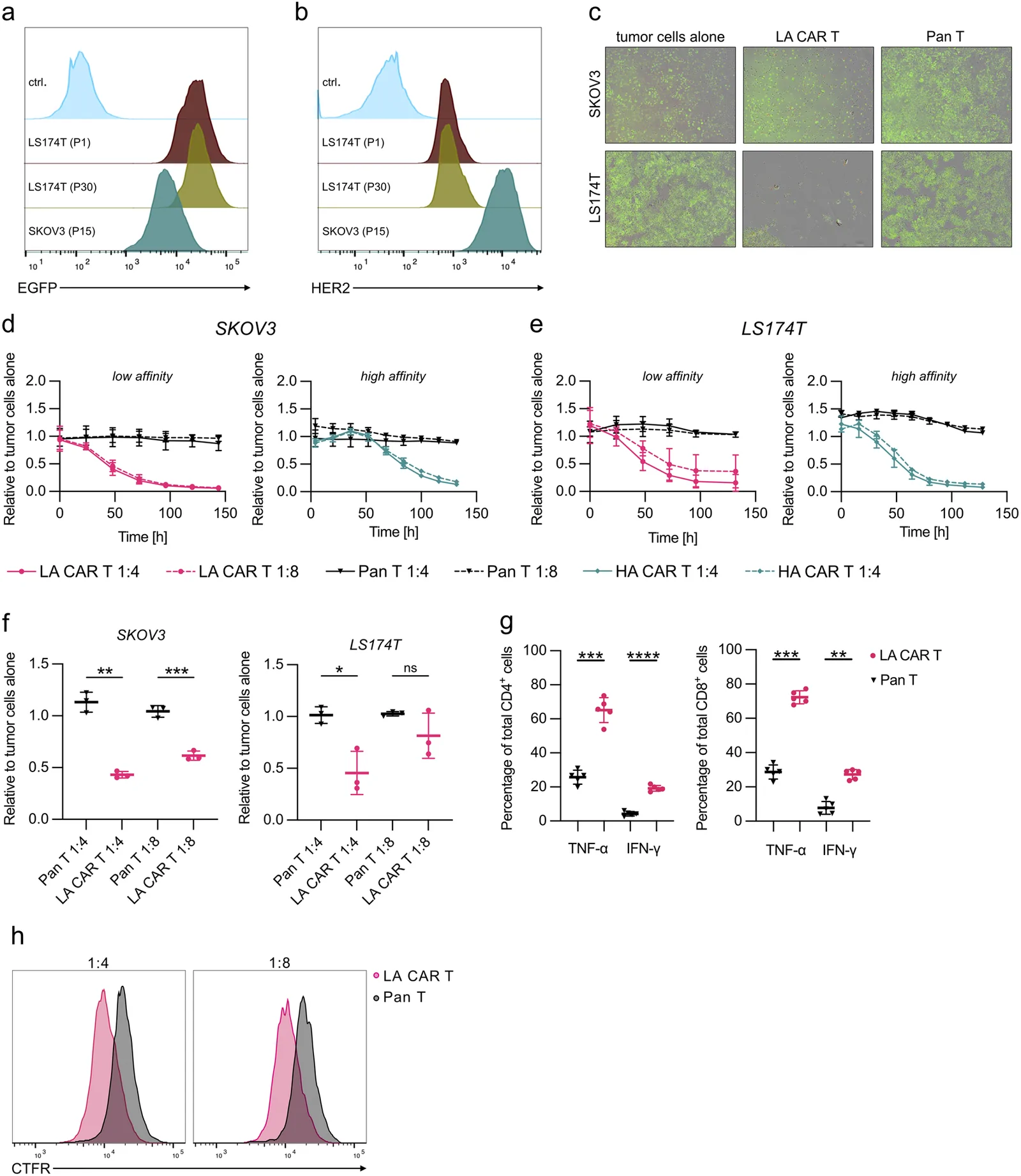
Sustained EGFP expression and enhanced Anti-HER2 CAR T cell cytotoxicity and activation. **(a)** EGFP expression of tumor cell lines was determined via flow cytometry analysis, demonstrating a sustained EGFP expression profile over at least 30 passages. **(b)** Flow cytometry analysis of HER2 expression of SKOV3 and LS174T cancer cells at different passages. **(c)** Representative phase contrast images of fluorescence-based killing assay illustrating a marked decrease in EGFP^+^ tumor cell count in the CAR T cell groups after 72 h (E:T 1:4). **(d,e)** Fluorescence-based comparison of antitumoral cytotoxicity between CAR T cells and Pan T cells at E:T ratios of 1:4 and 1:8. LA and HA Anti-HER2-CAR T cells exhibited more effective tumor cell killing against HER2^+^ SKOV3 and HER2^low^ LS174T cancer cells at both ratios (n = 4 for LA and n = 3 for HA Anti-HER2-CAR T cells). **(f)** Bioluminescence-based comparison of SKOV3 and LS174T tumor cell lysis between CAR T cells and Pan T cells. CAR T cells showed significantly enhanced cytotoxicity compared to control Pan T cells at different E:T ratios of 1:4 and 1:8 after co-culture for 72 h (n = 3). **(g)** IFN-γ and TNF-α expression by Pan T cells or CAR T cells. CAR T cells expressed significantly higher levels of IFN-γ and TNF-α compared to Pan T cells indicating stronger activation through HER2 stimulation via the CAR. **(h)** Comparative Analysis of CTFR-based Proliferation in Anti-HER2-CAR T Cells and Pan T Cells in co-culture with SKOV3 cells at E:T ratios of 1:4 and 1:8 after 7 days.

### Anti-HER2-CAR T cells demonstrate specific cytotoxicity against HER2^+^ and HER2^low^ cancer cells *in vitro*

Next, we investigated HER2 expression in SKOV3 ovarian cancer cells, a cell line demonstrating HER2 gene amplification and HER2^low^ LS174T CRC cells, that overexpress HER2 but do not harbor a gene amplification (Slamo et al., 1989; Tolmachev et al., 2007). As expected, mean fluorescence intensity (MFI)-based HER2 expression was about ten-fold higher in HER2^+^ SKOV3 cells, but considerably lower in HER2^low^ LS174T cells (Figure 1b, gating strategy in Supplementary Material 2).

To specifically target HER2 expressing cells, we used second generation LA Anti-HER2-CAR T cells that harbor an upstream myc-tag sequence, followed by the Anti-HER2 LA ScFv (4D5low), linked to a CD8 transmembrane and 4-1BB and CD3ζ intracellular signaling domains (Supplementary Material 1a).

When analyzing the antitumoral activity of LA Anti-HER2-CAR T cells against cancer cells in a fluorescence-based killing assay, LA Anti-HER2-CAR T cells demonstrated higher lysis than Pan T cells (i.e., non-transduced CD3^+^ cells isolated using the same isolation kit used for T cells that were then transduced with the CAR) at different effector-to-target (E:T) ratios (1:4; 1:8) (Figure 1c). Comparable antitumoral effects were also observed for HA Anti-HER2-CAR T cells (Figures 1d,e). Only Anti-HER2-CAR T cells showed abrogation of cancer cell growth compared to control Pan T cells after co-culture (n = 4 for LA, n = 3 for HA). Tumor cell lysis was observed at low E:T ratios: Even at the lowest E:T ratio (1:8) tested, Anti-HER2-CAR T cells almost completely eradicated cancer cells after 48 h. To further investigate the antitumoral activity of Anti-HER2-CAR T cells, we additionally performed bioluminescence-based killing assays (n = 3) (Karimi et al., 2014; Quah et al., 2012). When co-culturing LA Anti-HER2-CAR T cells with HER2^+^ SKOV3 or HER2^low^ LS174T cancer cells at different E:T ratios (1:4, 1:8), we found a significantly higher lysis in a bioluminescence based cytotoxicity test for Anti-HER2-CAR T cells compared to Pan T cells after 72 h (Figure 1f).

To further assess the activity of LA Anti-HER2-CAR T, we next performed flow cytometry (Gating strategy in Supplementary Material 4). We found that the cytokines IFN-γ and Tumor Necrosis Factor alpha (TNF-α) were significantly upregulated in Anti-HER2-CAR T cells compared to Pan T controls after coculture with HER2^+^ SKOV3 cells for 24 h (Figure 1g). Additionally, Anti-HER2 CAR T cells, but not Pan T cells showed proliferation after co-culture with cancer cells (Figure 1h).

In summary, these results highlighted the antitumoral efficacy of LA Anti-HER2-CAR T cells against HER2^+^ SKOV3 ovarian cancer and HER2^low^ LS174T CRC cells compared to Pan T cells.

### LA anti-HER2-CAR T cells effectively eradicate tumor cells in an *in vivo* model of systematically seeded HER2^+^ cancer

To analyze LA Anti-HER2-CAR T cell functionality *in vivo*, 1 × 10^6^ HER2^+^ SKOV3 ovarian cancer cells were injected i.v. into NXG mice, followed by (CAR) T cell in varying doses or PBS 7 days later (Figure 2a). Mice were monitored for tumor burden and systemic tumor seeding by weekly bioluminescence-based imaging. Before (CAR) T cell injection, all mice showed systemic tumor burden, primarily in the lungs. Total dosages of 0.1 × 10^6^ and 0.5 × 10^6^ Anti-HER2-CAR T cells were sufficient to eradicate tumor cells and control the systemic growth of HER2^+^ SKOV3/GFP-Luc ovarian cancer cells, thereby prolonging mice survival. One mouse injected with 0.1 × 10^6^ LA Anti-HER2-CAR T cells showed complete remission and survived until the predefined endpoint (Figures 2b,c). Further, we aimed to visualize systemic tumor seeding and burden and response to Anti-HER2-CAR T therapy. Bioluminescence imaging visualized response to LA Anti-HER2-CAR T with a reduction of bioluminescent pulmonary tumors and ultimately complete, durable remission following injection of 0.5 × 10^6^ LA Anti-HER2-CAR T cells (Figure 2c). All mice (n = 4) treated with 0.5 × 10^6^ LA Anti-HER2-CAR T cells survived until the predefined end of the experiment and showed significantly longer survival, and average radiance compared to mice that received 1 × 10^6^ untransduced T cells (Pan T cells) Figure 2d; weight curve in Supplementary Material 5). In contrast, PBS mice and Pan T mice had to be sacrificed between day 34 and 59 days and 45 and 59 days after T cell/PBS injection. To assess reproducibility, this experiment was independently repeated in two further, separate cohorts using the low-dose (0.1 × 10^6^) CAR T cell condition (combined n = 12 CAR T-treated and n = 6 Pan T-treated mice across all three cohorts), with consistent survival benefit observed in each (Supplementary Material 6).

**FIGURE 2:**
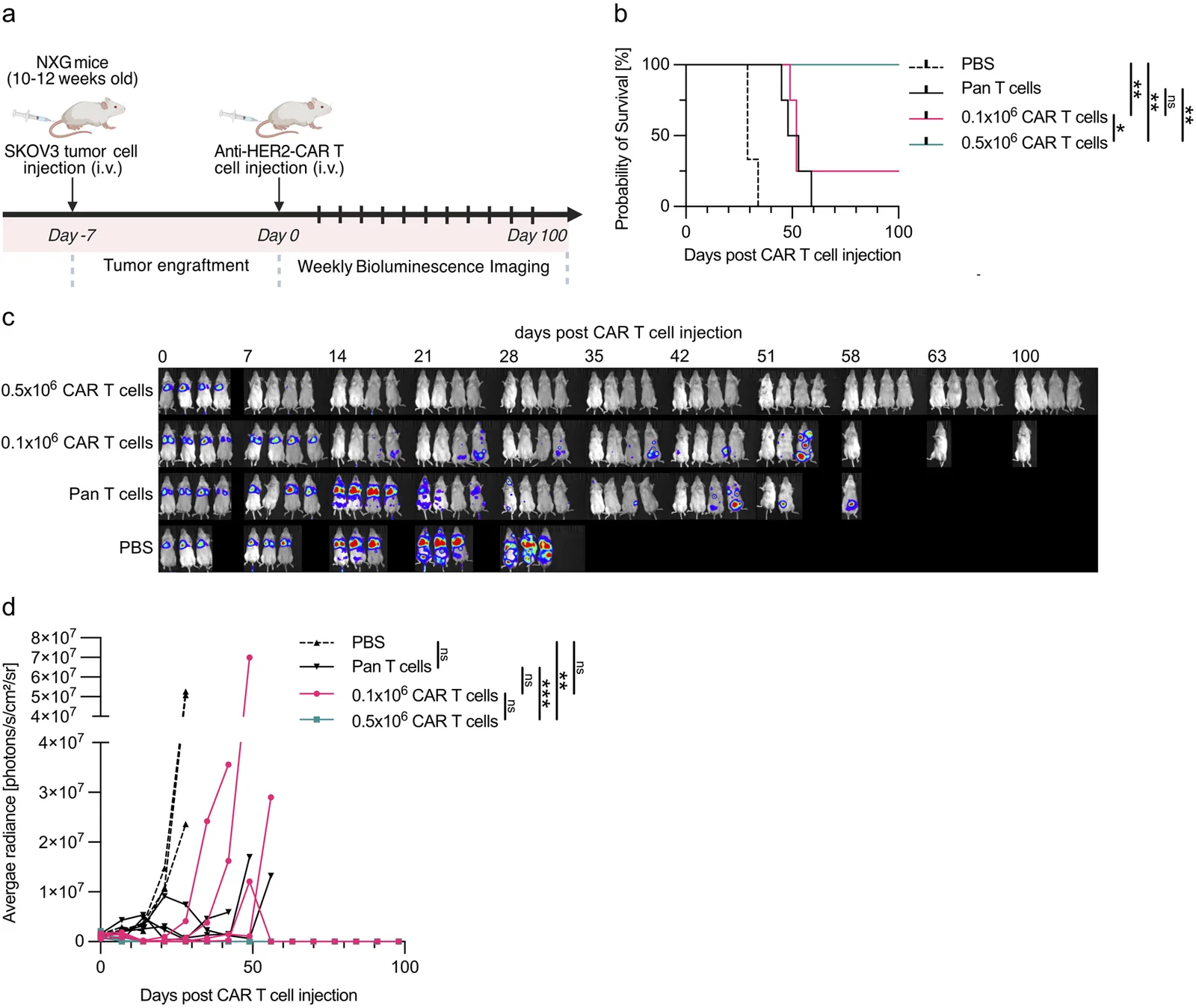
Dose-dependent *in vivo* efficacy of Anti-HER2 CAR T cells in a SKOV3 xenograft model. **(a)** Scheme of the xenograft cancer model. Immunodeficient female 10 to 12-week-old NOD*Prkdc* ^*scid*^ *-IL2rg* ^*Tm1*^/Rj were injected i.v. into the tail vein with 1 × 10^6^ HER2^+^ SKOV3/GFP-Luc 7 days prior to CAR T cell injection (day −7). On day 0, mice were injected i.v. into the tail vein with different doses of Anti-HER2-CAR T cells (0.1 × 10^6^; 0.5 × 10^6^). At the end of the experiment (100d after CAR T cell injection), mice were sacrificed. **(b)** Kaplan-Meier analysis of survival of mice injected with 1 × 10^6^ HER2^+^ SKOV3/GFP-Luc cancer cells (n = 4 mice per group). After injection of 0.5 × 10^6^ Anti-HER2-CAR T cells, mice survived significantly longer compared to mice treated with untransduced Pan T cells. A logrank Mantel-Cox test was used to test for statistical significance. **(c)** Bioluminescence imaging of mice injected with HER2^+^ SKOV3/GFP-Luc cancer cells and treated with Pan T cells and different dosages of Anti-HER2-CAR T cells. At indicated time points after T cell injection tumor burden was monitored by bioluminescence imaging. Bioluminescence images are shown for mice treated with 1 × 10^6^ Pan T, 0.1 × 10^6^ Anti-HER2-CAR T cells, 0.5 × 10^6^ Anti-HER2-CAR T cells (n = 4 per group), or PBS (n = 3). The dynamic range of the color code was set for each timepoint individually to compare the tumor burden between groups. **(d)** Bioluminescence based quantifications of tumor burden, measured by average radiance. Data were analyzed using Kruskal–Wallis test and Dunn’s multiple comparisons test.

Overall, we found that LA Anti-HER2-CAR T cells could effectively eradicate HER2^+^ SKOV3/GFP-Luc ovarian cancer cells even in an advanced systemic tumor state. Moreover, transduction of cancer cells with the novel bicistronic Luc2/EGFP vector reliably visualized systemic tumor seeding and burden utilizing bioluminescence imaging.

### LA anti-HER2-CAR T cells effectively inhibit tumor growth in an *in vivo* model of subcutaneous HER2^low^ cancer

LA Anti-HER2 CAR T cell efficacy was assessed in a subcutaneous *in vivo* model of HER2^low^ LS174T cells (Figure 3a). Consistent with tumor control, CAR T-treated mice achieved complete survival, whereas all animals in the Pan T and PBS group had to be euthanized according to pre-defined endpoints by day 32 (Figure 3b). Mice treated with 5 × 10^6^ LA Anti-HER2-CAR T cells exhibited tumor regression around day 14 and showed lasting tumor control throughout the 40-day observation period. In contrast, tumors in mice receiving 5 × 10^6^ Pan T cells or PBS continued to grow progressively (Figures 3c,d). Statistical analysis at day 18 confirmed significantly smaller tumor volumes in CAR T-treated mice relative to both Pan T cell and PBS groups.

**FIGURE 3:**
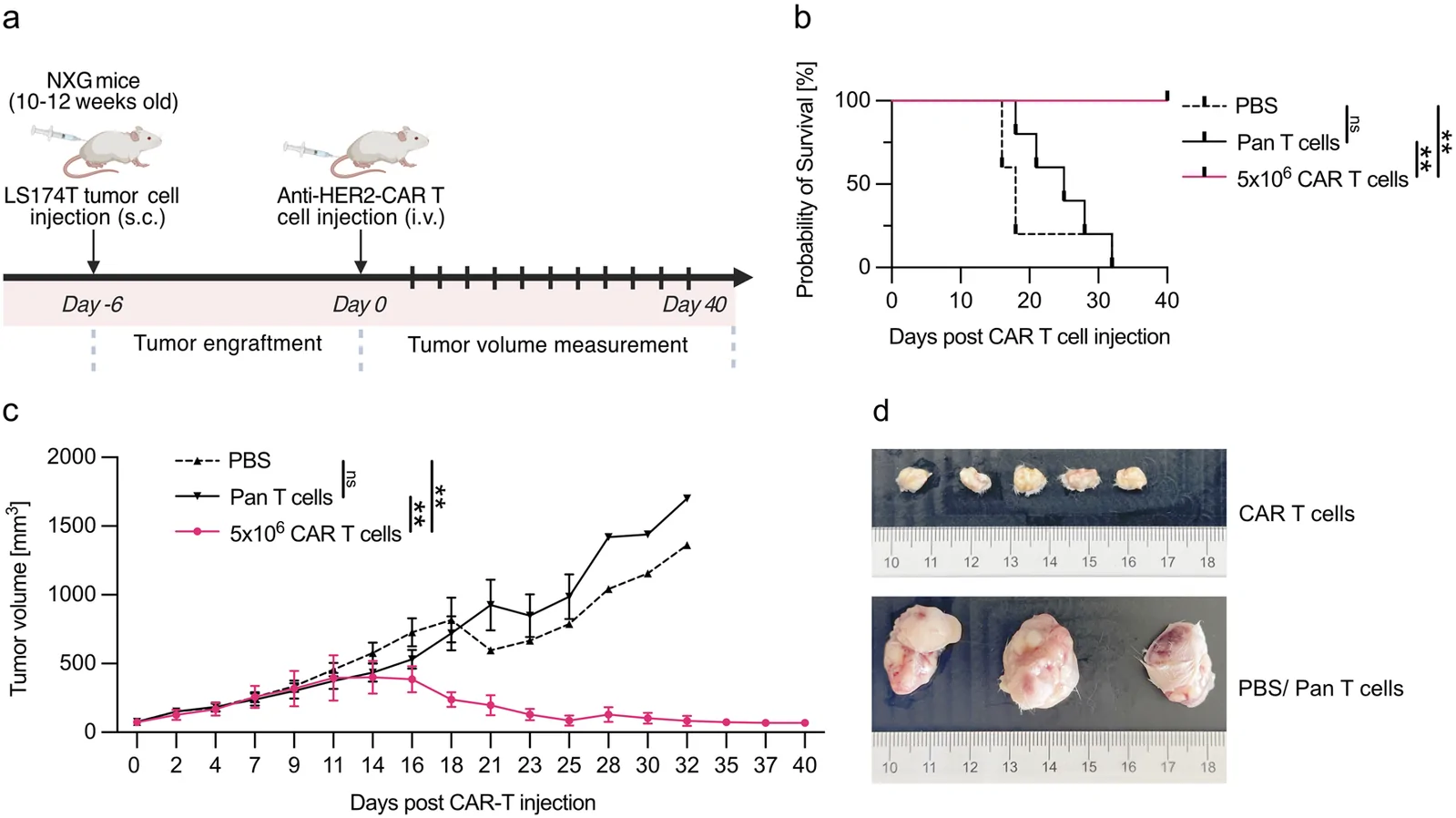
Anti-HER2 CAR T cell–mediated tumor control in a subcutaneous LS174T xenograft model. **(a)** Scheme of the s.c. cancer model. Immunodeficient female 10 to 12-week-old NOD-*Prkdc* ^*scid*^ *-IL2rg* ^*Tm1*^/Rj mice (Janvier Labs, France) were injected with 1.2 × 10^6^ LS174T cells subcutaneously in the flank (day −6). Once tumors were measurable, mice were randomized into equal groups based on tumor volume and injected with 5 × 10^6^ LA-Anti-HER2-CAR T cells i.v. into the tail vein (day 0). Treatment with an equal amount of untransduced Pan T cells or with PBS served as control. Mice were euthanized utilizing predefined humane end-points or 40 days after (CAR) T cell injection. **(b)** Kaplan–Meier survival analysis of mice treated with 5 × 10^6^ CAR T cells, 5 × 10^6^ Pan T cells. Survival was compared using the log-rank test. **(c)** Tumor volume (mean ± SD) following treatment. Data were analyzed using Kruskal–Wallis test and Dunn’s multiple comparisons test. **(d)** Representative images of explanted tumors from mice in the indicated treatment groups.

Collectively, these findings underline the promising antitumor efficacy and survival advantage conferred by LA Anti-HER2 CAR T cells in a HER2^low^ LS174T xenograft model.

### LA anti-HER2-CAR T cells effectively eradicate primary cancer organoids of HER2^+^ and HER2^low^ colorectal carcinoma while sparing healthy epithelial tissue

Next, we assessed the property of LA Anti-HER2-CAR T cells to discriminate between healthy human epithelium and HER2^low^ and HER2^+^ CRC organoids.

To this end, primary human CRC organoids from n = 3 patients and healthy colon organoids from n = 2 patients were generated. One CRC donor had HER2 gene amplification and a HER activating mutation, which was evident in panel sequencing and accompanied by strong HER2 overexpression (CRCO3). Two CRC donors had a HER2^low^ status based on IHC staining (n = 1 HER2 Score 1+, n = 1 HER2 Score 2+; Figure 4a). HER2 surface expression on CRC organoids in flow cytometry widely matched the clinical score and was comparable to HER2^low^ LS174T cells, lower than HER2 gene amplified SKOV3, but substantially higher compared to healthy colon organoids with physiological low-level HER2 expression (Figure 4b). Accordingly, both HA and LA Anti-HER2-CAR T cells showed cytolysis of HER2^+^ and HER2^low^ CRC organoids, but substantially reduced lysis of primary intestinal organoids (Figures 4c,d). Preservation of healthy organoids was trend-wise improved with LA CAR T cells relative to HA CAR T cells.

**FIGURE 4:**
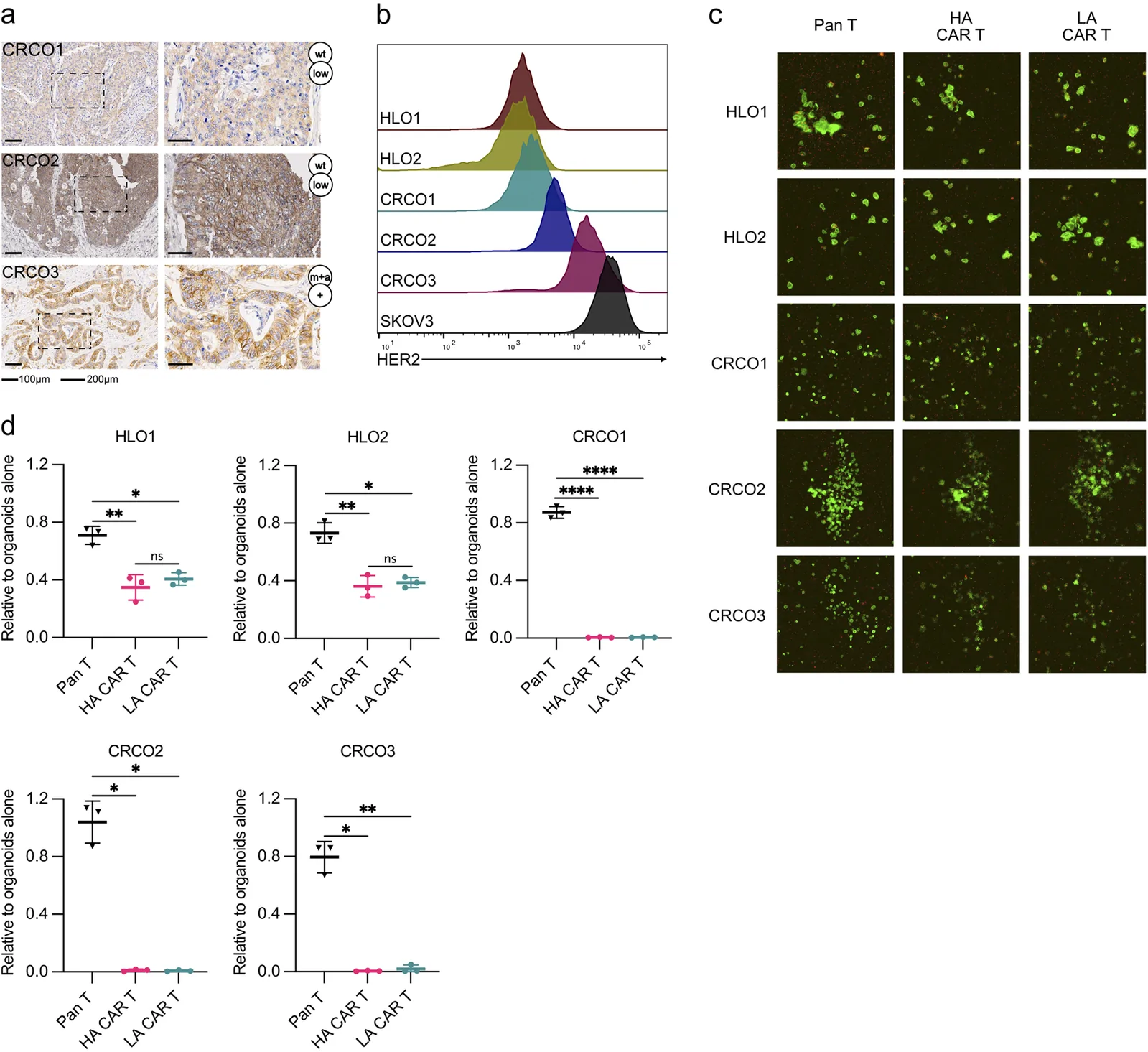
Selective targeting of HER2-expressing CRC organoids by Anti-HER2 CAR T cells. **(a)** Immunohistochemical (IHC) analysis in CRC organoids classified as HER2^low^ (low) for HER2 wild-type (wt) CRCO1 and CRCO2. CRCO3 demonstrates HER2 gene mutation and gene amplification (m+a) and is categorized as HER2^+^ (+). **(b)**. HER2 expression of CRC organoids (CRCO1-O3) and healthy human large intestinal organoids (HLO1 and HLO2) was determined via flow cytometry. **(c)** EGFP-expressing organoids were co-cultured with Pan T, LA or HA Anti-HER2-CAR T cells (E:T 1:2), with fluorescence microscopy images taken 24 h after coculture. **(d)** The number of viable cells in the organoids after 72 h of co-culture was determined using a luciferase assay, with values represented relative to the untreated organoid group. Anti-HER2-CAR T cells effectively targeted CRC organoids while displaying markedly reduced cytotoxicity against healthy intestinal organoids.

Collectively, these results underscore the capacity of the LA Anti-HER2-CAR T cells to discriminate between healthy epithelial tissues and HER2^low^ cancer.

## Discussion

While HA Anti-HER2-CAR T cell therapies have been associated with lethal side effects in pre-clinical models and first-in-human studies, LA Anti-HER2-CAR T cells have demonstrated efficacy against localized HER2^+^ cancers in pre-clinical studies (Morgan et al., 2010; Hernandez-Lopez et al., 2021; Shabaneh et al., 2024; Barden et al., 2024; Castellarin et al., 2020). Furthermore, LA murine Anti-HER2-CAR T cells have shown enhanced safety and reduced organ toxicity compared to their equivalents (Shabaneh et al., 2024; Castellarin et al., 2020). However, key questions remain: 1) how LA Anti-HER2 CAR T cells perform in the clinically relevant setting of systemic tumor stages, 2) whether they can still recognize HER2^low^ solid tumors while simultaneously demonstrating 3) low on-target off-tumor toxicities against healthy epithelial cells expressing physiological levels of HER2.

In this study, we aimed to evaluate the therapeutic effect of LA Anti-HER2 CAR T cells on these emerging obstacles and potentially extend the applicability of HER2-directed CAR T cell therapies to the wide range of HER2^low^ solid cancers entities that overexpress but do not amplify HER2.

We generated LA Anti-HER2-CAR T cells that effectively eradicated HER2^+^ SKOV3/GFP-Luc ovarian cancer cells and HER2^low^ LS174T CRC cells *in vitro*. Notably, LA Anti-HER2-CAR T cells achieved comparably potent tumor cell lysis at both E:T ratios tested, including the lowest ratio (1:8), indicating that even limited effector cell numbers were sufficient to mediate robust antitumor activity. This finding is consistent with efficient, repeated target-cell engagement and serial killing by activated CAR T cells. Supporting this activated phenotype, LA Anti-HER2-CAR T cells further showed elevated antigen-dependent cytokine production and proliferation upon encountering HER2+ cancer cells. This activated state was further confirmed by elevated antigen-dependent cytokine production and proliferation after encountering HER2^+^ cancer cells. In addition, LA Anti-HER2-CAR T cells halted tumor growth, induced complete remission, and improved survival rates *in vivo* even at low CAR T cell doses compared to mice that were treated with untransduced Pan T cells. Notably, the Anti-HER2-CAR used in this and related studies is directed against human HER2 and does not cross-react with the murine ortholog. Consequently, on-target off-tumor effects in murine xenograft models can only be meaningfully assessed using human tissue-derived systems rather than murine organ histopathology, a consideration that informed our experimental design. To address this limitation, we generated organoids from HER2^+^ and HER2^low^ CRC. Both LA and HA Anti-HER2-CAR T cells were able to clear CRC organoids, whereas LA Anti-HER2-CAR T cells demonstrated trend-wise reduced on-target off-tumor toxicity against healthy colon organoids compared to HA CAR T cells. Achieving differential activity against cancer cells and minimizing the risk of toxicity on vital organ systems such as the gastrointestinal tract is important to further improve CAR T outcomes. Besides the previously reported case of severe respiratory distress after infusion of Anti-HER2-CAR T cells, Feng et al. described one case of severe upper gastrointestinal hemorrhage in a phase I study of Anti-HER2 CAR T cells in patients with advanced-stage biliary tract or pancreatic cancer (Morgan et al., 2010; Feng et al., 2018). However, it is noteworthy that the patient had metastases in the gastric antrum (Feng et al., 2018). Overall, while the limited literature on gastrointestinal toxicities following Anti-HER2-CAR T cells precludes definitive conclusions due to varying study variables (e.g., CAR T cell dosages, scFv properties, CAR design, tumor location), gastrointestinal adverse effects are common, affecting over one-third of CAR T patients (Abu-Sbeih et al., 2019), and other HER2-directed therapies such as trastuzumab deruxtecan are associated with high rates of gastrointestinal complications (Guo et al., 2022). Therefore, the significantly reduced toxicity against healthy colon tissue represents a promising finding that remains to be corroborated in human trials.

CAR T cell therapy has demonstrated promising potential for hematological malignancies (Mehrabadi et al., 2022; Zhang et al., 2022; Cappell and Kochenderfer, 2023). Current research aims to establish CAR T cells as a treatment alternative in solid tumor entities and investigate the impact of CAR T cells on the systemic tumor burden (Guzman et al., 2023; Albelda, 2024). There is a myriad of syngeneic, xenograft, and transgenic murine CAR T models to help translate *in vitro* findings into clinical trials and improve solid tumor response to CAR T (Siegler and Wang, 2018). However, most models for solid cancers rely on the induction of local hetero- or orthotopic cancer manifestations. Yet, the primary cause of death in cancer patients is the systemic tumor burden, accounting for around 66% of cancer deaths in solid tumor patients (Siegler and Wang, 2018). Here, we aimed to simulate systemic tumor burden and track this phenotype in a murine model by establishing a high-fidelity luciferase system sensitive enough to detect systemically seeded tumors at early time points. Additionally, the vector created was also able to transduce primary CRC and healthy colon organoids. It allowed assessment of cell lysis based on bioluminescence and fluorescence signals also in primary material. This further enables sensitive *ex vivo* toxicity studies on healthy human epithelium that can provide insights into on-target off-tumor toxicity.

While CAR macrophages have already been shown to be effective against pulmonary cancer seeds, our study is the first to investigate the therapeutic potency of LA Anti-HER2-CAR T cells against the systemic tumor burden of HER2^+^ solid tumors (Sloas et al., 2021). The high sensitivity of *in vivo* bioluminescence imaging enabled reliable detection of tumor engraftment already at the earliest imaging timepoints in our model, consistent with previous reports demonstrating that this modality can detect even very low tumor cell numbers *in vivo*, including single tumor cells (Iwano et al., 2018). While alternative modalities such as microPET could offer complementary anatomical and quantitative information for future methodological work, bioluminescence imaging was selected in this study for its high sensitivity and suitability for longitudinal, repeated whole-body imaging without the usage of radiotracers. Our approach leveraged low CAR T dosages (0.5 × 10^6^ cells) to mediate complete systemic tumor remission *in vivo*. This finding is particularly relevant in the context of the dose-dependent toxicity associated with CAR T cell therapy, as lower CAR T dosages (i.e., <1 × 10^8^ cells/m^2^) have been demonstrated to reduce the risk of severe therapy-induced side effects such as the Cytokine Release Syndrome, whereas higher dosages are associated with increased risk (Ahmed et al., 2015; Morgan et al., 2010; Li et al., 2020; Wagner et al., 2020; Ahmed et al., 2017; Haas et al., 2023). Treatment related adverse effects were monitored over an observation period of 100 days, which represents a substantial portion of the feasible lifespan of immunodeficient NXG mice. Given the lack of cross-reactivity between our human HER2-directed CAR and the murine antigen, longer-term murine toxicity data would in any case be of limited translational value, underscoring the importance of our human organoid-based toxicity assessment and future clinical evaluation.

Our study also demonstrated promising therapeutic effects of LA Anti-HER2-CAR T cells in a subcutaneous HER2^low^ *in vivo* model. Previous research showed that subcutaneous tumor models present with additional therapeutic challenges, such as reduced perfusion, increased hypoxia and altered tumor microenvironment, compared to orthotopic models (Brand et al., 2020; Zhang et al., 2019). Consequently, circulating CAR T cells may have difficulty extravasating and distributing into the tumor sites (Liu et al., 2021), which might be further complicated by the low HER2 expression in our subcutaneous model. In addition, hypoxia has been demonstrated to reduce ATP production in CAR T, with impaired CAR T metabolism showing poor antitumoral effects (Hatae et al., 2024). Similarly, hypoxic conditions have been implicated with upregulation of immunosuppressive genes (e.g., *CD274*), which are well-known to impair antitumoral CAR T effects (Gumber and Wang, 2022; Ouyang et al., 2024). Overall, overcoming these challenges underlines the therapeutic potency of LA Anti-HER2-CAR T cells in the context of systemic tumor burden and reflects a translational strength, demonstrating activity under clinically representative, immunosuppressive conditions. In the subcutaneous HER2^low^ model, LA Anti-HER2-CAR T cells also showed no obvious side-effects (e.g., organ failure) until the predefined endpoints. While the current literature on therapy-induced side-effects of CAR T cells in subcutaneous *in vivo* models remains limited (Ramos-Cardona et al., 2022), our findings suggest a favourable efficacy-safety profile, warranting future larger-scale mechanistic and translational work.

Anti-HER2-CAR T cells have already been investigated for different cancer entities (Ahmed et al., 2015; Li et al., 2020; Ahmed et al., 2017; Xu et al., 2021; Song et al., 2018; Forsberg et al., 2019; Shen et al., 2019; Murayama et al., 2021; Rosewell et al., 2017). However, their clinical application is limited, mainly due to on-target-off-tumor toxicity towards healthy epithelial cells (Morgan et al., 2010). Furthermore, rapid Anti-HER2-CAR T activation and exhaustion after antigen encounter in healthy tissues is associated with poor infiltration into tumors and antitumor activity, as recently demonstrated in a murine model (Shabaneh et al., 2024). While findings on on-target off-tumor toxicity in pre-clinical models remain to be interpreted in light of their limited transferability to human patients, they may still guide future research and point toward potential risks (Van Nor and man, 2019). One possible approach to overcome on-target off-tumor toxicity and overactivation of CAR T cells is to increase the threshold for CAR T cell activation by reducing the scFv affinity towards the target antigen through mutations (Hernandez-Lopez et al., 2021; Shabaneh et al., 2024). The previously characterized scFv variants used in this study (4D5low for LA, parental 4D5 for HA) demonstrated a substantially reduced binding affinity of the low-affinity variant compared to its high-affinity counterpart (Hernandez-Lopez et al., 2021), providing a well-defined biophysical basis for the differential activation threshold underlying our approach. Consistent with our human data, murine LA Anti-HER2-CAR T cells also showed superior safety and reduced on-target off-tumor effects in a pre-clinical model of murine HER2^+^ tumors (Shabaneh et al., 2024). However, previous research did not investigate if LA Anti-HER2-CAR T cells would still be able to dissect HER2^low^ malignancies from healthy epithelium without the genetic background of HER2 gene amplification. HER2^+^ tumors occur around 15-fold less frequently than HER2^low^ malignancies that account for ∼40% of solid epithelial tumors (Yan et al., 2015; Uzunparmak et al., 2023). In our analyses, we demonstrated that HER2^low^ cancer cell lines and CRC organoids indeed could be effectively eradicated by LA Anti-HER2-CAR T cells, while healthy epithelial cells expressing physiological HER2 levels were protected from CAR T cell-based killing.

Overall, our LA Anti-HER2-CAR T cells may provide a novel strategy to combat systemic tumor burden in HER2^low^ solid cancers. This line of research may introduce CAR T therapy as a possible treatment for high-prevalence cancers.

## Conclusion and limitations

In conclusion, we generated functional LA Anti-HER2-CAR T cells that exhibited effective antitumor activity with reduced toxicity against healthy epithelial cells. Systemic cancer burden as well as *ex vivo* cancer and healthy epithelial organoids could be assessed by a novel vector enabling highly sensitive bioluminescence and fluorescence-based quantifications. Our results suggest that LA Anti-HER2-CAR T cells may provide a novel treatment option for HER2^+^ and HER2^low^ tumors.

However, our preclinical study also confers limitations: While effective discrimination between physiological and supraphysiological antigen expression is essential for minimizing on-target off-tumor toxicities, both potent differential cytotoxicity and minimal off-tumor side effects remain challenging to model in pre-clinical studies. In particular, while our organoid model captures epithelial HER2 expression in a patient-derived, physiologically relevant format, it does not recapitulate full tissue architecture, stromal components, or an intact tumor microenvironment, all of which could modulate on-target off-tumor toxicity *in vivo*. Furthermore, *in vivo* efficacy was assessed using SKOV3 and LS174T cell line xenografts; while these findings were complemented by primary patient-derived CRC organoids from three independent donors spanning a range of HER2low and HER2-amplified status, extension of this work to additional HER2-expressing solid tumor entities, such as gastric or breast cancer models, represents an important direction for future studies to further explore the generalizability of these findings. Additionally, HA Anti-HER2-CAR T cells were not included in the *in vivo* experiments, given their previously reported association with severe on-target off-tumor effects in preclinical and first-in-human studies (Morgan et al., 2010; Castellarin et al., 2020); a direct *in vivo* head-to-head comparison between LA and HA Anti-HER2-CAR T cells was therefore not conducted. Further insights from clinical trials are pivotal to assess patient safety following the administration of LA Anti-HER2-CAR T cells.

## Methods

### Lentivirus production

HEK293T cells were used to generate pantropic VSV-G pseudotyped second generation lentivirus via multi-plasmid transfection. Cells were prepared at a confluence of 80%–90% and plated at a density of 3 × 10^5^ cells/mL in 10 mL complete medium on tissue culture-treated 10 cm plates. The cells were evenly distributed by gentle swirling and then incubated overnight. Before transfection (1 h), the medium was replaced with fresh antibiotic-free DMEM with 10% FBS. Lentivirus was generated by lipid-mediated transient transfection in HEK293T cells using the Lipofectamine 3000 Kit (Thermo Fisher Scientific, United States). Each plate was transfected with 10 µg of packaging plasmid psPAX2 (Addgene plasmid #12260; http://n2t.net/addgene:12260; RRID: Addgene_12260), 5 µg of envelope plasmid pMD2.G (Addgene plasmid #12259; http://n2t.net/addgene:12259; RRID: Addgene _12259), and 10 µg of transfer plasmid harboring the gene of interest. Transfer plasmids pHR_SFFv_4D5-LowCAR_RHL001, encoding for the LA Anti-HER2-CAR, and pHR_SFFv_4D5-WT-Highest-CAR_RHL004, encoding for the LA Anti-HER2-CAR, were a gift from Wendell Lim (LA: Addgene plasmid #164824; http://n2t.net/addgene:164824; RRID: Addgene_164824; HA: Addgene plasmid #164826; https://www.addgene.org/164826/; RRID: Addgene_164826). The binding kinetics of both scFv variants were previously characterized by surface plasmon resonance, with the low-affinity 4D5low scFv showing a substantially reduced binding affinity compared to the parental high-affinity 4D5 scFv (Kd = 210 nM vs. 1.9 nM, respectively) (Hernandez-Lopez et al., 2021).

Transfer plasmid Luc2-P2A-NLS-EGFP (Vector builder accession number VB2203141064msu) was used for generation of GFP/Luc Lentivirus. Plasmids were isolated using Pure Link HiPure Plasmid Midiprep Kit (Thermo Fisher Scientific, United States) (Hernandez-Lopez et al., 2021). Twenty-four hours after transfection, the medium was changed and cells were incubated for another 24 h before collecting the supernatant. Collected supernatant was centrifuged at 1,000 g for 10 min at RT to remove cell debris. Supernatant was transferred into a 50 mL syringe and filtered through a 0.45 μm membrane filter to further clear cell debris. Next, 38 mL of the supernatant was transferred into Ultra-Clear Centrifuge tubes (Beckman Coulter, United States). Ultracentrifugation was performed at 50.000 rcf for 120 min at 4 °C using the SW 32 Ti Swinging-Bucket rotor in the Optima XE-90 ultracentrifuge (Beckman Coulter, United States) and supernatant was discarded. Each pellet was resuspended in 200 μL PBS (1x). Aliquots of the concentrated virus were stored at −80 °C. Functional viral titers were determined using HEK293T cells.

### Cell culture

Cells were cultivated in a moisture-saturated environment containing 5% CO_2_ at 37 °C. Adherent cell types were grown in conventional tissue culture-treated T75 flasks, while cells in suspension were cultivated in standard, untreated T75 and T25 flasks. DMEM enriched with 10% FBS (PAN-Biotech, Germany), and 1% Penicillin/Streptomycin (P/S; Thermo Fisher, United States) (DMEM Basic medium) was used for culturing HEK293T. For transfection with lentiviral plasmids, HEK293T cells were plated in Corning® 100 mm tissue culture-treated plates (Corning Incorporated, United States) with DMEM containing 10% FBS, excluding antibiotics. SKOV3/GFP-Luc and LS174T/GFP-Luc cells were cultured in RPMI-1640 medium with 10% FBS (PAN-Biotech, Germany), 1% P/S (Thermo Fisher, United States), 1% Gibco™ GlutaMAX™ (200 mM) (Thermo Fisher, United States), 1% Gibco™ HEPES (1M) (Thermo Fisher, United States), Gibco™ 1% sodium pyruvate (100 mM) (Thermo Fisher, United States), and 1% of a 100x non-essential amino acids solution (PAN-Biotech, Germany) (CAR T medium). (CAR-) T cells were maintained in the same medium supplemented with IL-7 (5 × 10^7^ U/mL) and IL-15 (5 × 10^6^ U/mL) (Miltenyi, Germany).

### T cell isolation and activation

Healthy voluntary donor peripheral blood mononuclear cells (PBMCs) were obtained from the Department of Transfusion Medicine, University Hospital Regensburg, after informed written consent under the ethic vote number 21-2224-101 Regensburg. PBMCs were isolated using density gradient centrifugation (PancollTM Cell Separation Medium, PAN-Biotech, Germany) and negative magnetic cell separation using (MACSprep™ Pan T Isolation Kit, Miltenyi, Germany). Following T cell isolation, T Cell TransAct™ (Miltenyi, Germany) was added to the cells (1:100) to activate T cells.

### Lentiviral T cell transduction

The isolated Pan T cells were transduced with the lentivirus encoding the Anti-HER2-CAR 3 days after the activation: 1.5 × 10^6^ lentiviral particles in 500 µL growth medium were centrifuged to a RetroNectin (Takara Bio, Japan) coated 24-well plate at 32 °C and 1500g for 2 h. Next, the viral supernatant was removed from the wells. 1 × 10^6^ activated T cells were suspended in 1 mL medium with IL-7 and IL-15 per well and centrifuged to the plate at 320g for 5 min (MOI = 1.5). The following day, the transduced T cells were harvested from the plate, and the transduction efficiency of Anti-HER2-CAR T cells was evaluated using flow cytometry by staining for the myc tag included in the extracellular CAR domain (Miltenyi anti-myc FITC, 130-116-653, 1:100).

### Cell lysis assays

The killing efficacy of Anti-HER2-CAR T cells was determined using bioluminescence and fluorescence-based assays. The Incucyte® S3 Live-Cell Analysis Instrument (Sartorius, Germany) was utilized to analyze cancer cell killing through fluorescent-based live-cell imaging. In coculture assays, 1 × 10^5^ SKOV3/GFP-Luc or LS174T/GFP-Luc cells per well were seeded in 24-well plates 6 h prior to CAR T cell addition. Subsequently, Anti-HER2-CAR T or Pan T cells were added to the plates at different E:T ratios as indicated and cancer cell count was analysed based on GFP-positive nuclei using the Incucyte software (Version v2020C, Sartorius, Germany). Data is presented as the green object count of different conditions normalized to tumor cells alone, which served as the baseline control. For bioluminescence-based cytolysis assays, a Spark® microplate reader (Tecan Life Sciences, Switzerland) was used. 2.5 × 10^4^ cells (SKOV3) were seeded to white 96-well plates before adding CAR T cells at different E:T ratios as indicated. At the indicated time-points, 50 µL IVISbrite™ D-Luciferin (26.7 mg/mL) (Revvity, Germany) was added, cells were incubated at 37 °C for 15 min and luminescence was measured.

### Flow cytometry analysis

1 × 10^5^ SKOV3/GFP-Luc cancer cells per well were seeded in 24-well plates 6 h prior to the start of the experiment. Subsequently, Anti-HER2-CAR T or Pan T cells were added to the plates at different E:T ratios as indicated. (CAR) T cells were collected after 24 h and were labeled with Zombie UV (1:300) for live/dead staining. Phenotype was assessed with anti-human CD3 BV786 (1:100), anti-human CD4 BV711 (1:100), anti-human CD8a BUV805 (1:100), and anti-myc HER2 CAR PE/FITC (1:200). Cells were fixed, permeabilized, and labeled with anti-human IFN-γ BB700 (1:100) and anti-human TNF-α BUV395 (1:100) for intracellular staining. For flow cytometry analysis, cells were processed as described above and were analyzed by a BD FACSSymphony™ flow cytometer (BD Biosciences, United States). Data were analyzed with FlowJo v10.10 (BD Biosciences, United States). More details on the antibodies are shown in Supplementary Material 7.

### Cell trace far red proliferation assay

Anti-HER2-CAR T cells or Pan T cells were labeled the CellTrace™ Far Red proliferation kit (Thermo Fisher, United States) following the manufacturer’s instructions. The stained cells were the co-cultured with SKOV3 cells at E:T ratios of 1:4 and 1:8. After 7 days, Cell Trace Far Red (CTFR) expression in Anti-HER2-CAR T cells and Pan T cells was analyzed using flow cytometry. A reduction in CTFR intensity directly corresponds to increased cell proliferation, since proliferation dye is diluted with each cell division. This approach allows the evaluation of the proliferation of Anti-HER2-CAR T cells and Pan T cells in response to SKOV3 cells, based on changes in CTFR fluorescence levels.

### Animal experiments

In the i.v. xenograft cancer model, immunodeficient female 10- to 12-week-old NOD-*Prkdc* ^*scid*^ *IL2rg* ^*Tm1*^/Rj (Janvier Labs, France) were injected i.v. into the tail vein with 1 × 10^6^ HER2^+^ SKOV3/EGFP-Luc 7 days prior to (CAR) T cell injection (day −7). On day 0, mice were randomized into equal groups based on bioluminescence measurements. To evaluate the effect of LA Anti-HER2-CAR T cells, increasing doses of LA Anti-HER2-CAR T cells were injected i.v. into the tail vein. Treatment with 0.5 × 10^6^ untransduced Pan T cells served as control. Mice were scored daily. Tumor progression, systemic seeding, and response to CAR T therapy was confirmed regularly by bioluminescence imaging using a Xenogen IVIS imaging system (PerkinElmer, United States) after i.p. injection of 200 µL IVISbrite D-Luciferin (15 mg/mL, Revvity; United States), and the intensity of the signal was measured as average radiance (photon/second/cm^2^/steradian [p/s/cm^2^/sr]) and total flux (photons/second [photons/s]). Mice were euthanized utilizing humane end-points or 100 days after (CAR) T cell injection.

In the LS174T subcutaneous xenograft cancer Immunodeficient female 10 to 12-week-old NOD-*Prkdc* ^*scid*^ *-IL2rg* ^*Tm1*^/Rj mice (Janvier Labs, France) were injected subcutaneously with 1.2 × 10^6^ LS174T cells suspended in 0.2 mL of a 50% Matrigel Basement Membrane Matrix/PBS solution in the flank (day −6). Once tumors were measurable using an electronic caliper, mice were randomized into equal groups based on tumor volume and injected with 5 × 10^6^ LA-Anti-HER2-CAR T cells i.v. into the tail vein (day 0). Treatment with an equal amount of untransduced Pan T cells or with PBS served as control. The longest (length, L) and the perpendicular shortest (width, W) external diameters of the tumors were measured three times weekly until study termination using an electronic caliper. Tumor volume was calculated according to the standard modified ellipsoid formula (V = 0,5 × L × W^2^). Data are presented as mean ± SD (n = 5). For all procedures requiring anesthesia, mice were anesthetized with inhalational isoflurane delivered via a precision vaporizer (RWD Life Science, China). Induction was performed with 3%–5% isoflurane in 100% oxygen at 0.8–1.0 L/min; maintenance with 1.5%–2.5% isoflurane in 100% oxygen at 0.8–1.0 L/min. Depth of anesthesia was monitored by assessing the pedal withdrawal and corneal reflexes and was adjusted as necessary to maintain an adequate plane of anesthesia. At the study endpoint or upon reaching predefined humane endpoints, mice were anesthetized with isoflurane and euthanized by cervical dislocation performed by trained personnel in accordance with institutional animal care and use guidelines. All mouse experiments were approved by the Animal Ethics Committee of University Hospital Regensburg and the Animal Ethics Committee of Regierung Unterfranken (Animal Test Proposal Number: RUF-55.2.2-2532-2–1,563) and performed in accordance with relevant institutional and national guidelines and regulations.

### Organoid generation and maintenance

The study was approved by the local ethics boards (458/17 S (Amendment: 256/18 S) at Phillips University Marburg and 14-47_2–101 at Technical University Munich and #14-47_2101 at UKR). All patients gave informed, written consent to Colon biopsies were washed with PBS and digested with collagenase (2 mg/mL, Sigma-Aldrich, United States) for 25 min at 37 °C. The digested tissue was filtered through a 100 μm cell strainer, and the resulting cell suspension was centrifuged to collect a pellet. The pellet was resuspended in Matrigel and seeded into 24-well plates. After the Matrigel solidified, IntestiCult™ Organoid Growth Medium (STEMCELL Technologies, Canada) was added to each well. Human colon organoids were passaged weekly and maintained in organoid growth medium. To generate GFP-Luc-labeled organoids, the organoids were digested into single cells and transduced with a lentiviral vector encoding bicistronic expression of Luc2 and nuclear EGFP. After 5 days of culture, the organoids were digested into single cells again, and GFP-positive cells were sorted using fluorescence-activated cell sorting. All co-culture experiments were performed using organoids within a maximum of five passages after thawing of freshly-frozen organoids post-generation, specifically to minimize passage-related phenotypic drift, including potential changes in HER2 or transgene expression.

### Organoid and anti-HER2-CAR T cell co-culture

Organoids were cultured in organoid growth medium until day 6 post-passage. To estimate the number of cells per drop of organoid culture, one drop was digested into single cells. Intact organoids were released from Matrigel using Cell Recovery Solution (Corning, United States).

Anti-HER2-CAR T cells and control Pan T cells, labeled with CellTrace™ Far Red (ThermoFisher, United States), were counted and prepared at an E:T ratio of 2:1. Organoids and T cells were mixed in a co-culture medium composed of 10% Matrigel, 45% organoid growth medium, and 45% T cell medium. Images were taken at 24 h post-co-culture using the Incucyte® S3 Live-Cell Analysis Instrument (Sartorius, Germany). After 72 h of co-culture, the viability and metabolic activity of the organoid cells were assessed using a luciferase assay as described previously.

### Statistical analysis

Data were analyzed as mean ± SD by paired and unpaired t-tests. If not otherwise indicated, one-way ANOVA and Tukey *post hoc* test were performed for multiple comparisons. Survival curve was analyzed by Kaplan–Meier curves and log-rank test. Significance was set at p values <0.05, p < 0.01, p < 0.001 and p < 0.0001 (*, **, ***, and ****). All experiments were repeated at least three times unless otherwise stated. Non-parametric tests (Kruskal–Wallis with Dunn’s multiple comparisons test) were applied for *in vivo* tumor burden and tumor volume comparisons, as normality of the underlying distributions could not be reliably assumed given the small per-group sample sizes typical of *in vivo* xenograft studies.All statistical analyses were performed with GraphPad Prism v10.2.0 (GraphPad Prism, United States).

## Data Availability

The original contributions presented in the study are included in the article/Supplementary Material, further inquiries can be directed to the corresponding authors.

## Supplementary material

SUPPLEMENTARY MATERIAL 1 Design of the LA Anti-HER2 CAR and Luc2-NLS-EGFP tracking vector **(a)** Anti-HER2-CAR construct. Schematic diagram of the LA Anti-HER2-CAR, consisting of an upstream c-myc-tag sequence, followed by the Anti-HER2 LA scFv domain, linked to CD8 transmembrane domain and 4-1BB and CD3ζ intracellular signaling domains. The 4D5low ScFv binding affinity was characterized before and has a substantially lower k_d_ towards the HER2 antigen compared to the parental 4D5 ScFv (210 nM vs. 1.9 nM) (Hernandez-Lopez et al., 2021). **(b)** Luc2-P2A-NLS-EGFP vector VB220314-1064msu. To sensitively track both cancer cell viability and cell count *in vivo* and *in vitro*, we constructed a VSV-G pseudotyped lentiviral vector encoding for both, a humanized high-fidelity firefly luciferase (Luc2) and enhanced green fluorescent protein coupled to nuclear localization sequences at both ends (NLS-EGFP) driven by the strong, constitutively expressed Human eukaryotic translation elongation factor 1 α1 (EF1A) promoter.

SUPPLEMENTARY MATERIAL 2 Gating strategy for analysis of EGFP and HER2 expression of tumor cells Gating strategy for flow cytometry analysis of EGFP and HER2 expression of SKOV3 and LS174T cells.

SUPPLEMENTARY MATERIAL 3 Transduction efficiency, viability and expansion of Anti-HER2 CAR T cells. **(a)** Transduction efficiency of CAR T cells **(b)** Viability of Anti-HER2-CAR T cells at 0, 3, 7, and 10 days after transduction for n = 5 healthy voluntary donors. (**c)** Mean expansion of Anti-HER2-CAR T cells at 0, 3, 7,10, and 14 days after transduction for n = 5 healthy voluntary donors.

SUPPLEMENTARY MATERIAL 4 Flow cytometry gating of CAR and Pan T cell subsets Representative flow cytometry gating strategy for the identification of cell subsets in the Anti-HER2-CAR T and Pan T cell population.

SUPPLEMENTARY MATERIAL 5 Mouse body weight curves across treatment groups Relative weights of mice treated with 0.1 × 10^6^ Anti-HER2 CAR T cells (n = 4), 0.5 × 10^6^ Anti-HER2 CAR T cells (n = 4), Pan T cells (n = 4), and PBS (n = 3). All values are shown as mean ± SD (error bars).

SUPPLEMENTARY MATERIAL 6 Low-dose Anti-HER2 CAR T cells improve survival and reduce tumor burden Overall, 0.1 × 10^6^ CAR T cells confer a significant survival benefit and durable responses compared to 0.1 × 10^6^ Pan T cells. **(a)** Scheme of the xenograft cancer model. Immunodeficient female 10 to 12-week-old NOD-*Prkdc* ^*scid*^ *-IL2rg* ^*Tm1*^/Rj (Janvier Labs, France) were injected i.v. into the tail vein with 1 × 10^6^ HER2^+^ SKOV3/GFP-Luc 7 days prior to CAR T cell injection (day −7). On day 0, mice were injected i.v. into the tail vein with 0.1 × 10^6^ Anti-HER2-CAR T cells from two different donors in two independent experiments. At the end of the experiment (100d after CAR T cell injection), mice were sacrificed. **(b)** Bioluminescence imaging of mice injected with HER2^+^ SKOV3/GFP-Luc cancer cells and treated with Pan T cells and Anti-HER2-CAR T cells (n = 6 mice for Pan T group, n = 12 mice for CAR T group). At indicated time points after T cell injection tumor burden was monitored by bioluminescence imaging. The dynamic range of the color code was set for each timepoint individually to compare the tumor burden between groups. **(c)** Kaplan-Meier analysis of survival of mice injected with 1 × 10^6^ HER2^+^ SKOV3/GFP-Luc cancer cells. After injection of 0.1 × 10^6^ Anti-HER2-CAR T cells, mice survived significantly longer compared to mice treated with untransduced Pan T cells. A logrank Mantel-Cox test was used to test for statistical significance. **(d)** Bioluminescence-based quantifications of tumor burden, measured by total flux.

## Glossary

ANOVA: Analysis of Variance
CAR: Chimeric Antigen Receptor
CD: Cluster of Differentiation markers
CRC: Colorectal Cancer
CTFR: Cell Trace Far Red
DMEM: Dulbecco’s Modified Eagle Medium
E:T: Effector-to-Target
FITC: Fluorescein Isothiocyanate
GFP: Green Fluorescent Protein
GFP-Luc: Green Fluorescent Protein-Luciferase
HER2: Human Epidermal growth factor Receptor 2
IFN-γ: Interferon gamma
IL: Interleukin
i.v.: Intravenous
Luc: Luciferase
MACS: Magnetic-Activated Cell Sorting
MOI: Multiplicity of Infection
NSG: NOD-*Cg-Prkdcˢ* ^*cid*^ *Il2rg^tm^* ^*1*^ *^wjl^/SzJ*
PBMCs: Peripheral Blood Mononuclear Cells
PBS: Phosphate Buffered Saline
PET: Positron Emission Tomography
P/S: Penicillin/Streptomycin
RT: Room Temperature
RPMI: Roswell Park Memorial Institute Medium
s.c.: Subcutaneous
SD: Standard Deviation
SKOV3/GFP-Luc: SKOV3 cells expressing Green Fluorescent Protein and Luciferase
SPF: Specific-Pathogen-Free
TNF-α: Tumor Necrosis Factor alpha
VSV-G: Vesicular Stomatitis Virus G Protein
