# Supplementary material for: Low-affinity anti-HER2-CAR T cells efficiently eradicate HER2+ and HER2low cancer cells with reduced toxicity on healthy tissue
Source: Front Bioeng Biotechnol. 2026 Oct 12;14:1895507. doi: 10.3389/fbioe.2026.1895507 (PMC13696918; doi:10.3389/fbioe.2026.1895507)
Supplement: Supplementary file 1 [file Supplementaryfile1.pdf]

SUPPLEMENTAL MATERIAL

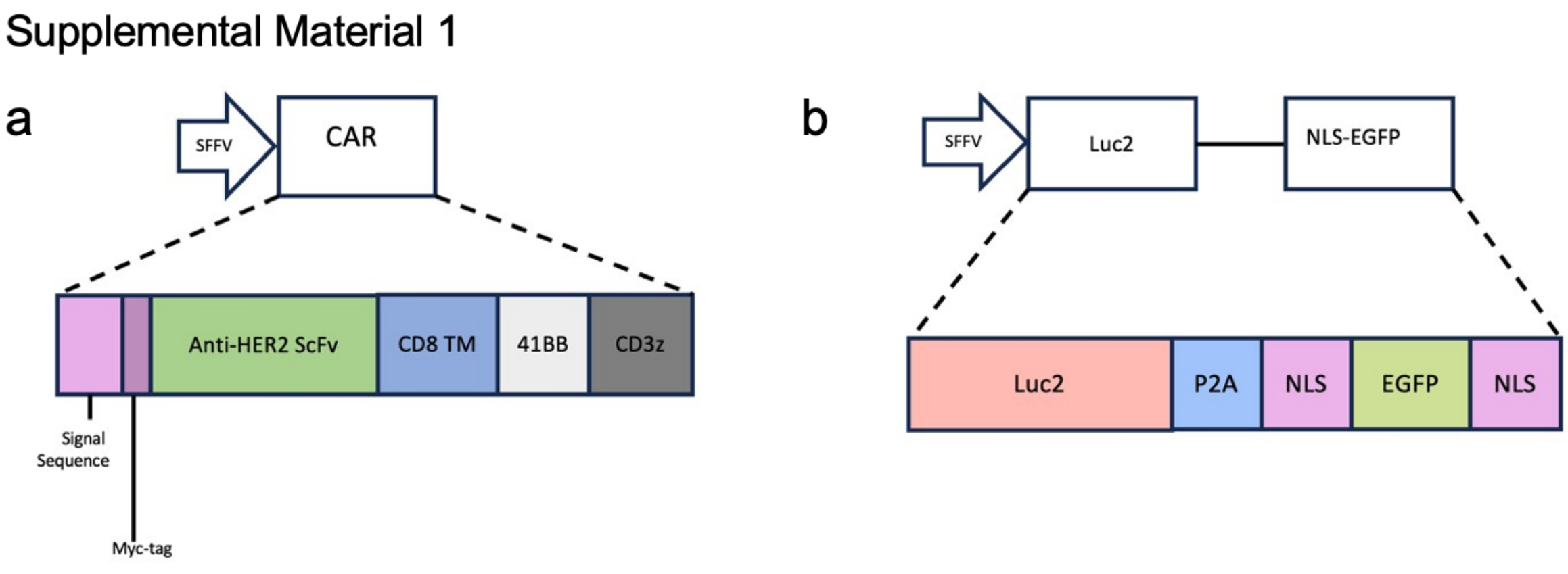

Supplemental Material 1. Design of the LA Anti-HER2 CAR and Luc2-NLS-EGFP tracking vector

(a) Anti-HER2-CAR construct. Schematic diagram of the LA Anti-HER2-CAR, consisting of an upstream c-myc-tag sequence, followed by the Anti-HER2 LA scFv domain, linked to CD8 transmembrane domain and 4-1BB and CD3ζ intracellular signaling domains. The 4D5low ScFv binding affinity was characterized before and has a substantially lower k_d towards the HER2 antigen compared to the parental 4D5 ScFv (210nM vs. 1.9nM) (20). (**b)** Luc2-P2A-NLS-EGFP vector VB220314-1064msu. To sensitively track both cancer cell viability and cell count *in vivo* and *in vitro*, we constructed a VSV-G pseudotyped lentiviral vector encoding for both, a humanized high-fidelity firefly luciferase (Luc2) and enhanced green fluorescent protein coupled to nuclear localization sequences at both ends (NLS-EGFP) driven by the strong, constitutively expressed Human eukaryotic translation elongation factor 1 α1 (EF1A) promoter.

Supplemental Material 2

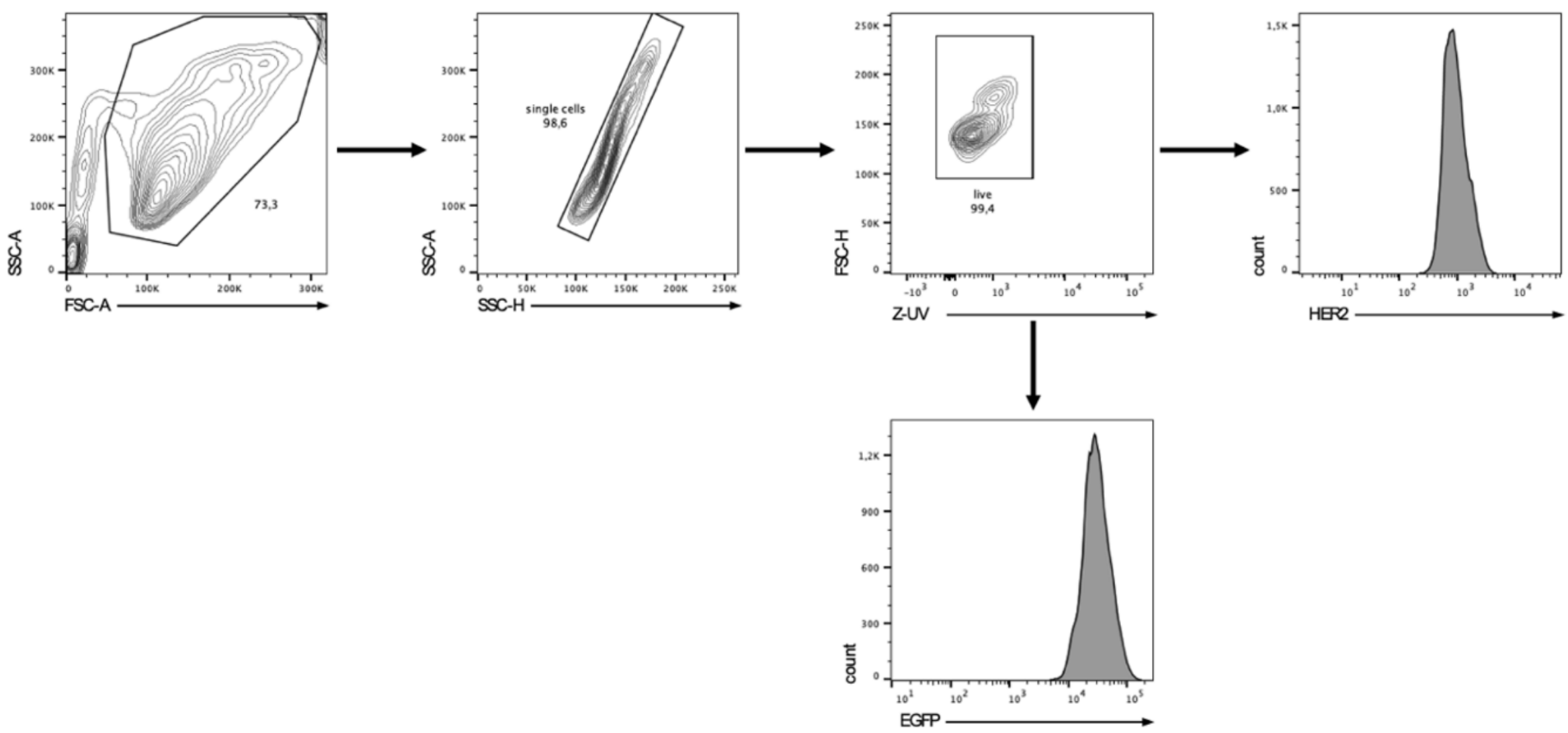

Supplemental Material 2. Gating strategy for analysis of EGFP and HER2 expression of tumor cells

Gating strategy for flow cytometry analysis of EGFP and HER2 expression of SKOV3 and LS174T cells.

Supplemental Material 3

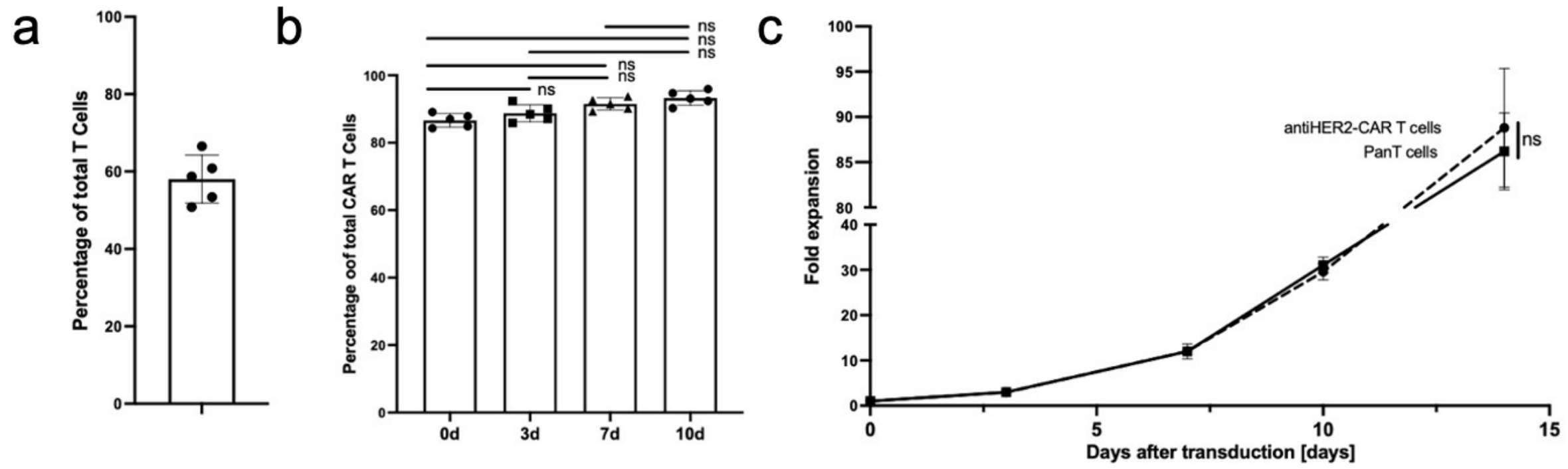

Supplemental Material 3. Transduction efficiency, viability and expansion of Anti-HER2 CAR T cells

(a) Transduction efficiency of CAR T cells (**b)** Viability of Anti-HER2-CAR T cells at 0, 3, 7, and 10 days after transduction for n=5 healthy voluntary donors. (**c)** Mean expansion of Anti-HER2-CAR T cells at 0, 3, 7,10, and 14 days after transduction for n=5 healthy voluntary donors.

Supplemental Material 4

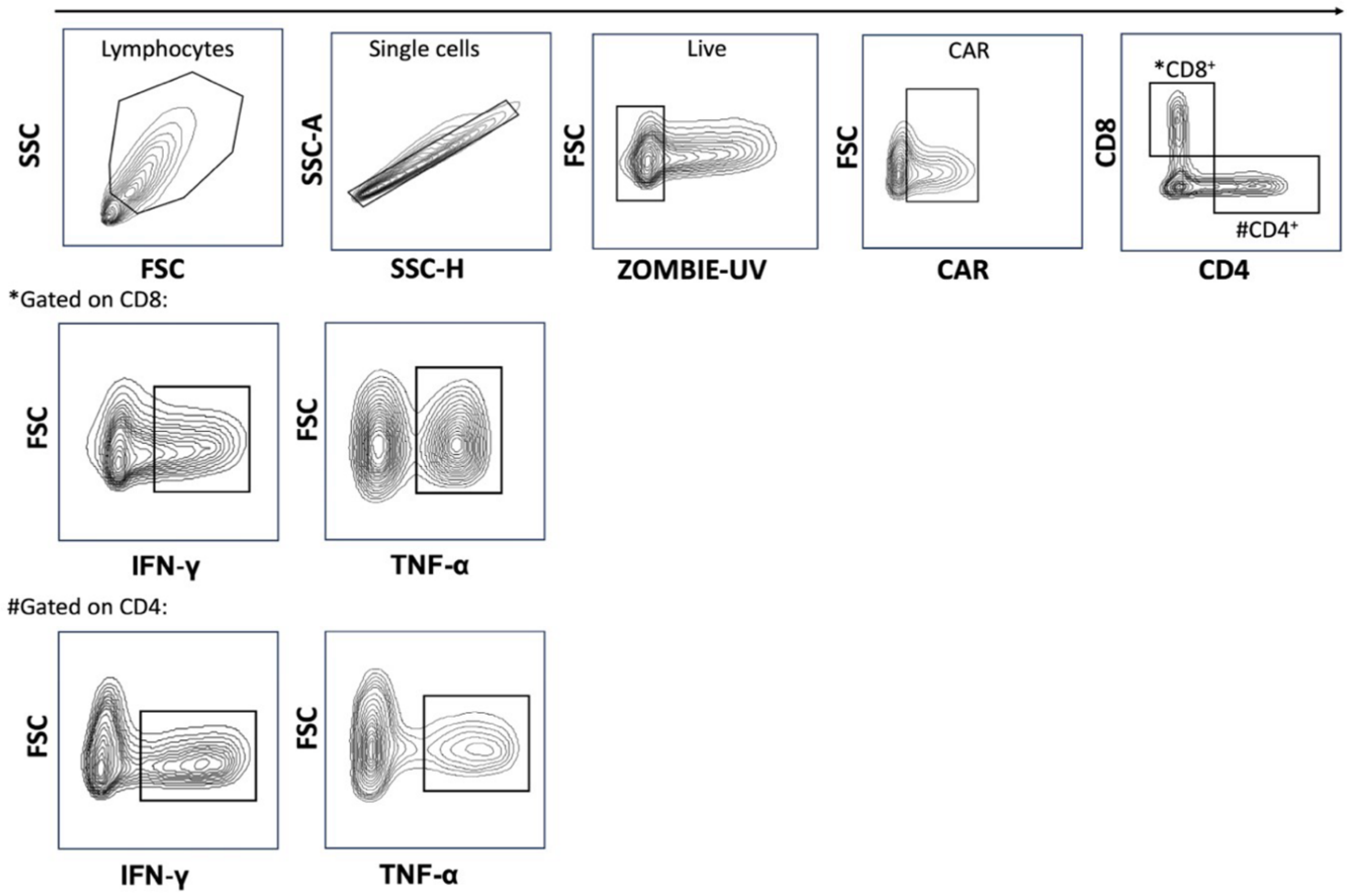

Supplemental Material 4. Flow cytometry gating of CAR and Pan T cell subsets

Representative flow cytometry gating strategy for the identification of cell subsets in the Anti-HER2-CAR T and Pan T cell population.

Supplemental Material 5

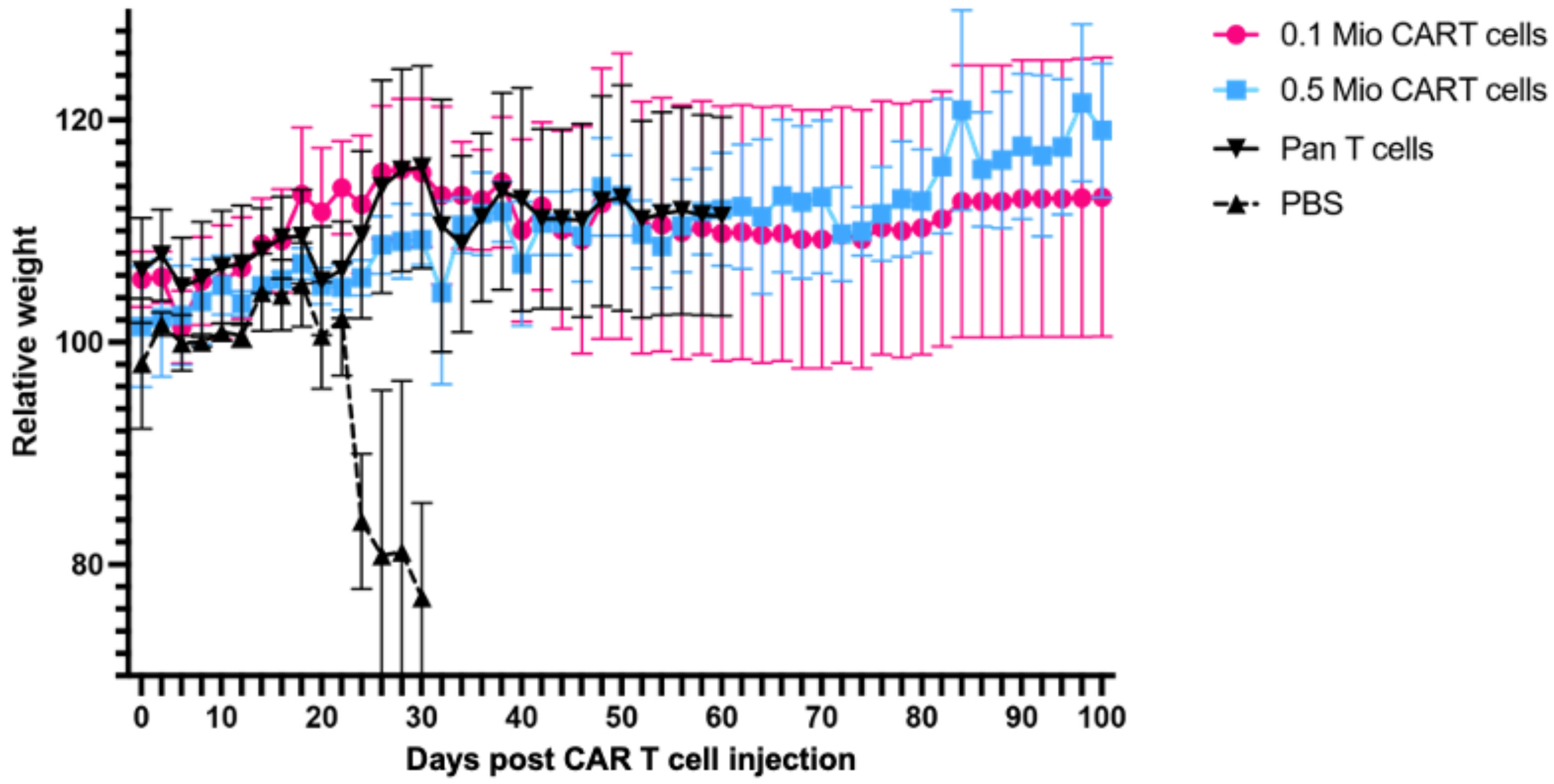

Supplemental Material 5. Mouse body weight curves across treatment groups

Relative weights of mice treated with 0.1×10^6 Anti-HER2 CAR T cells (n=4), 0.5×10^6 Anti-HER2 CAR T cells (n=4), Pan T cells (n=4), and PBS (n=3). All values are shown as mean ± SD (error bars).

Supplemental Material 6

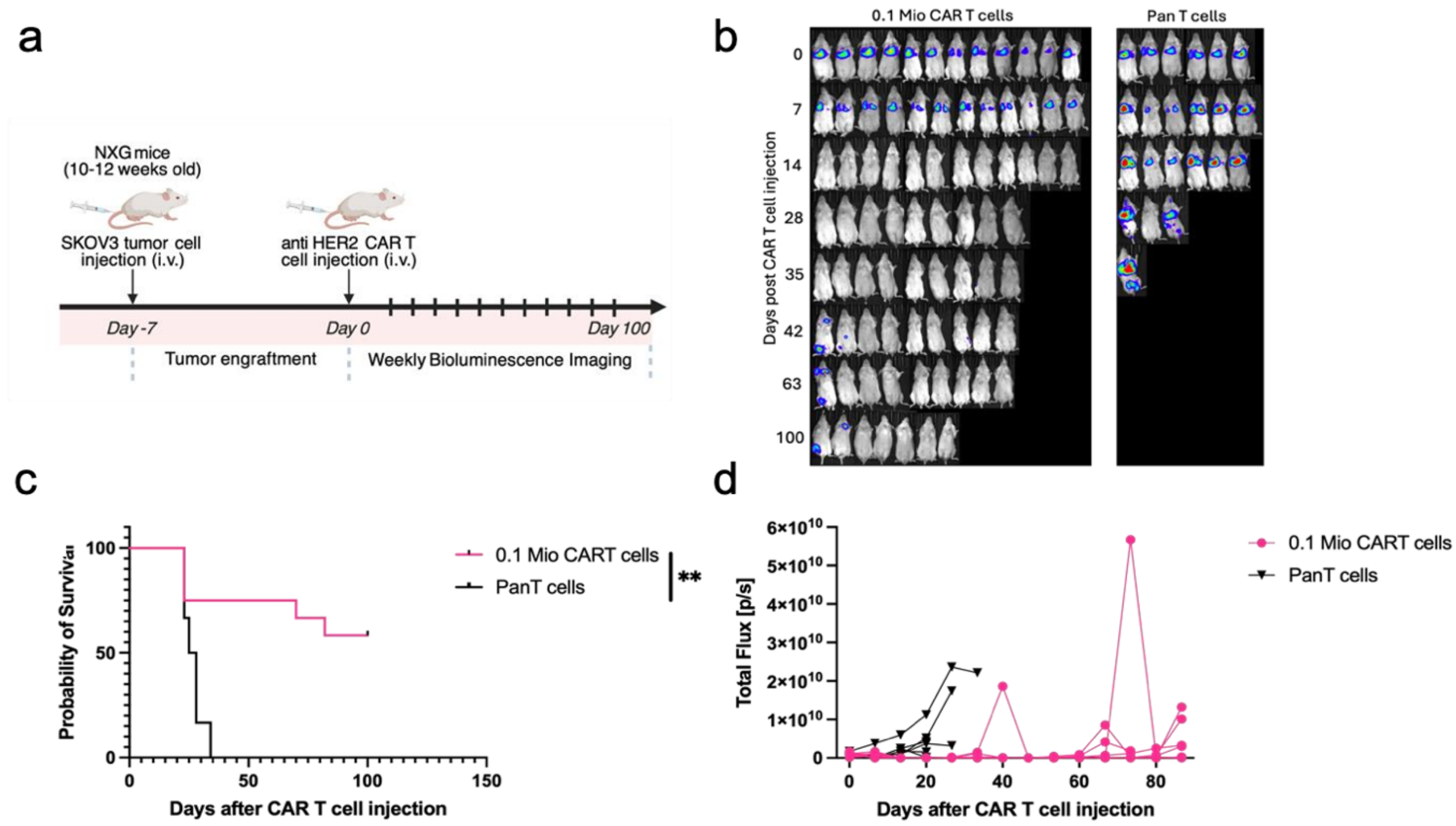

Supplemental Material 6. Low-dose Anti-HER2 CAR T cells improve survival and reduce tumor burden

Overall, 0.1×10^6 CAR T cells confer a significant survival benefit and durable responses compared to 0.1×10^6 Pan T cells. **(a)** Scheme of the xenograft cancer model. Immunodeficient female 10 to 12-week-old NOD-*Prkdc*^{scid}-*IL2rg*^{Tm1}/Rj (Janvier Labs, France) were injected i.v. into the tail vein with 1×10^6 HER2$^+$ SKOV3/GFP-Luc 7 days prior to CAR T cell injection (day -7). On day 0, mice were injected i.v. into the tail vein with 0.1×10^6 Anti-HER2-CAR T cells from two different donors in two independent experiments. At the end of the experiment (100d after CAR T cell injection), mice were sacrificed. **(b)** Bioluminescence imaging of mice injected with HER2$^+$ SKOV3/GFP-Luc cancer cells and treated with Pan T cells and Anti-HER2-CAR T cells (n=6 mice for Pan T group, n=12 mice for CAR T group). At indicated time points after T cell injection tumor burden was monitored by bioluminescence imaging. The dynamic range of the color code was set for each timepoint individually to compare the tumor burden between groups. **(c)** Kaplan–Meier analysis of survival of mice injected with 1×10^6 HER2$^+$ SKOV3/GFP-Luc cancer cells. After injection of 0.1×10^6 Anti-HER2-CAR T cells, mice survived significantly longer compared to mice treated with untransduced Pan T cells. A logrank Mantel-Cox test was used to test for statistical significance. **(d)** Bioluminescence-based quantifications of tumor burden, measured by total flux.

Supplemental Material 7

List of antibodies used for flow cytometry analysis:

PE anti-human CD340 (erbB2/HER-2) Antibody (BioLegend, #324405)

Brilliant Violet 421™ anti-human CD3 Antibody (BioLegend, #300433)

Brilliant Violet 510™ anti-human CD4 Antibody (BioLegend, #317443)

Brilliant Violet 605™ anti-human CD8 Antibody (BioLegend, #344741)

Zombie UV™ Fixable Viability Kit (BioLegend, #423107) PE

Streptavidin (BioLegend, #405203)

c-myc Antibody, anti-human/mouse/rat, Biotin (Miltenyi Biotec #130-124-899)

BUV395 Mouse Anti-Human TNF-α, (BD, #563996)

BB700 Mouse Anti-Human IFN-γ, (BD, # 566394)
