# Supplementary material for: Low-affinity anti-HER2-CAR T cells efficiently eradicate HER2+ and HER2low cancer cells with reduced toxicity on healthy tissue
Source: Front Bioeng Biotechnol. 2026 Oct 12;14:1895507. doi: 10.3389/fbioe.2026.1895507 (PMC13696918; doi:10.3389/fbioe.2026.1895507)
Supplement: Supplementary file 2 [file Supplementaryfile2.pdf]

Supplemental Material 7

List of antibodies used for flow cytometry analysis:

PE anti-human CD340 (erbB2/HER-2) Antibody (BioLegend, #324405)

Brilliant Violet 421™ anti-human CD3 Antibody (BioLegend, #300433)

Brilliant Violet 510™ anti-human CD4 Antibody (BioLegend, #317443)

Brilliant Violet 605™ anti-human CD8 Antibody (BioLegend, #344741)

Zombie UV™ Fixable Viability Kit (BioLegend, #423107) PE Streptavidin (BioLegend, #405203)

c-myc Antibody, anti-human/mouse/rat, Biotin (Miltenyi Biotec #130-124-899)

BUV395 Mouse Anti-Human TNF-α, (BD, #563996)

BB700 Mouse Anti-Human IFN-γ, (BD, # 566394)
